# Lipoxin A4 receptor agonist BML-111 induces autophagy in alveolar macrophages and protects from acute lung injury by activating MAPK signaling

**DOI:** 10.1186/s12931-018-0937-2

**Published:** 2018-12-05

**Authors:** Huaizheng Liu, Kefu Zhou, Liangkan Liao, Tianyi Zhang, Mingshi Yang, Chuanzheng Sun

**Affiliations:** Emergency and Intensive Care Center, The Third Xiangya Hospital of Central South University, 138 Tongzipo Road, Changsha, 410013 Hunan Province, PR China

**Keywords:** Acute lung injury, BML-111, Autophagy, Lipoxin, Apoptosis

## Abstract

**Background:**

Acute lung injury (ALI) is a life-threatening lung disease where alveolar macrophages (AMs) play a central role both in the early phase to initiate inflammatory responses and in the late phase to promote tissue repair. In this study, we examined whether BML-111, a lipoxin A4 receptor agonist, could alter the phenotypes of AM and thus present prophylactic benefits for ALI.

**Methods:**

In vitro, isolated AMs were treated with lipopolysaccharide (LPS) to induce ALI. In response to BML-111 pre-treatment, apoptosis and autophagy of AMs were examined by flow cytometry, and by measuring biomarkers for each process. The potential involvement of MAPK1 and mTOR signaling pathway was analyzed. In vivo, an LPS-induced septic ALI model was established in rats and the preventative significance of BML-111 was assessed. On the cellular and molecular levels, the pro-inflammatory cytokines TNF-α and IL-6 from bronchoalveolar lavage were measured by ELISA, and the autophagy in AMs examined using Western blot.

**Results:**

BML-111 inhibited apoptosis and induced autophagy of AMs in response to ALI inducer, LPS. The enhancement of autophagy was mediated through the suppression of MAPK1 and MAPK8 signaling, but independent of mTOR signaling. In vivo, BML-111 pre-treatment significantly alleviated LPS-induced ALI, which was associated with the reduction of apoptosis, the dampened production of pro-inflammatory cytokines in the lung tissue, as well as the increase of autophagy of AMs.

**Conclusions:**

This study reveals the prophylactic significance of BML-111 in ALI and the underlying mechanism: by targeting the MAPK signaling but not mTOR pathway, BML-111 stimulates autophagy in AMs, attenuates the LPS-induced cell apoptosis, and promotes the resolution of ALI.

## Introduction

Acute lung injury and acute respiratory distress syndrome (ALI/ARDS) are life-threatening, diffuse lung injuries triggered by various lung pathologies such as pneumonia, sepsis, and ischemia-reperfusion, and presenting a mortality of approximately 40% [1]. The pathological progression of ALI/ARDS involves an acute phase featuring the rapid release of pro-inflammatory cytokines including tumor necrosis factor α (TNFα), interleukin 1β (IL-1β), IL-6, and type I interferon (IFN) followed by edema and infiltration of neutrophils, macrophages, and red blood cells into alveoli, impairing alveolar functions; an ensuing subacute phase characterized by proliferation of alveolar type II cells and interstitial fibrosis; and an ending chronic phase represented by the resolution of acute edema/inflammation and tissue repair, with or without exacerbated fibrosis that indicates incomplete or complete resolution, respectively [2]. Alleviating inflammatory damage and promoting complete tissue repair are keys to ALI treatment.

Alveolar macrophages (AMs) are phagocytes localized in the lung tissue and essential for defense against harmful pathogenic microbes. During the acute phase of ALI, AMs are activated, release cytokines and chemokines to stimulate neutrophil infiltration, and initiate pulmonary inflammation (M1 phenotype) [3]. Later on, however, these cells adopt an alternative anti-inflammatory M2 phenotype and promote tissue repair [4]. Intensive efforts are dedicated to understanding the mechanisms regulating macrophage phenotypes and functions during ALI development, which will benefit the treatment and improve the outcome of ALI. Among the various mechanisms explored, autophagy critically regulates macrophage functions on multiple levels: from their generation, recruitment, differentiation, to polarization [5]. Autophagy is a biological process whereby cells survive nutrient limitation by degrading cytoplasmic components in lysosomes for maintaining energy homeostasis [6]. Two signaling molecules critically controls the initiation of autophagy, AMP-activated protein kinase (AMPK) that activates and mammalian target of rapamycin (mTOR) that inhibits autophagy [7]. Autophagy is executed through the formation of autophagosomes, which involves the conversion of cytosolic LC3-I to LC3-phosphatidylethanolamine conjugate (LC3-II), and thus the LC3-II/LC3–1 ratio is frequently used as a quantitative indicator for autophagy [8]. In addition to LC3, Beclin 1 (BECN1) and SQSTM1/p62, up-regulated and reduced during autophagy, respectively, are also functionally important and frequently measured as markers for autophagy [9, 10]. Functionally, autophagy may promote or protect from apoptosis of AMs, depending on the disease paradigms and/or microenvironmental stimuli [11, 12]. However, minimal is known how autophagy is regulated during ALI development and whether it is functionally beneficial or detrimental to ALI progression.

Lipoxins (LXs) are endogenous lipids synthesized from arachidonic acid pathways by immune cells such as macrophages and neutrophils, and well demonstrated for their anti-inflammatory and pro-resolving activities [13]. Four lipoxins have been identified so far, LXA4, LXB4, 15-epi-LXA4 and 15-epi-LXB4_._ The anti-inflammatory activities of LXs are mediated through the G-protein-coupled LXA4 receptor, followed by distinct signaling cascades and transcription factors [13]. Cumulative evidence suggests that LXs attenuate lung injury by acting on multiple cell types, including macrophages, epithelial cells, and endothelial cells [14, 15], although the underlying mechanisms are not well understood. Consistently, studies show that stable LX analogs and LXA4 receptor agonists present potent anti-inflammatory activities and may benefit inflammatory diseases [13, 16, 17].

A recent study showed that 15-epi-LXA4 stimulated the autophagy of macrophages by activating MAPK1, independent of mTOR signaling, and as a functional consequence, promoted phagocytosis of these cells [18]. However, it is not known whether the same mechanism may also bring any benefits to ALI. To answer this question, we established an in vitro as well as an in vivo lipopolysaccharide (LPS)-induced sepsis-associated ALI model, specifically examined the biological effects of pre-treating cells with LXA4 receptor agonist, BML-111 on the apoptosis and autophagy of AMs, explored the underlying signaling mechanisms, and assessed the prophylactic potential of BML-111 in ALI. Here we showed that BML-111, by targeting MAPK signaling but not mTOR signaling, stimulates autophagy and inhibits apoptosis in AMs, alleviating ALI-associated inflammation and tissue injury.

## Materials and methods

### Isolation of AMs from rats

All animal experiments in this study were approved by the Institutional Animal Care and Use Committee, Center for Medical Ethics, Central South University (Changsha, China). Male Sprague Dawley rats with an average weight between 200 and 250 g were purchased from Hunan SJA Laboratory Animal Co., Ltd. (Changsha, China) and housed in a specific pathogen-free facility at room temperature of (22 ± 1)°C on a 12/12-h light/dark cycle, with access to food and water ad libitum. The isolation of AMs was performed as described previously [19]. Upon isolation, these cells were cultured in DMEM medium (Gibco, Carlsbad, CA, USA) at 37 °C in humidified atmosphere of 5% CO_2_. To induce ALI-related damage, isolated AMs were treated with vehicle (PBS), LPS (*Escherichia coli* serotype 055:B5, 1 μg/mL; Sigma, St. Louis, MO, USA), BML-111 (100 nM; Cayman Chemical, Ann Arbor, MI, USA). AMs were treated with BML-111 for 6 h prior to LPS treatment for a further 2 h. MHY1485 was purchased from MCE (10 μM; MedChem Express, NJ, USA). The autophagy inhibitor, chloroquine and the mTOR inhibitor, rapamycin were purchased from MedChem Express (Monmouth Junction, NJ, USA) and administered to cells at the final concentrations of 0.5 μM and 20 μg/mL, respectively.

### 3-(4,5-dimethylthiazol-2-yl)-2,5-diphenyltetrazolium bromide (MTT) assay for cell viability

Isolated AMs were seeded into 96-well plates (Corning, Corning, NY, USA) in triplicate at 1 × 10^4^ cells/100 μL/well at 37 °C in a humidified 5% CO_2_ incubator. After treating the cells with vehicle, LPS, BML-111, or LPS + BML-111 for 24 h, 20 μL of MTT agent (5 mg/mL) was added into each well and incubated at 37 °C for a further 4 h. After gentle shaking and removal of the supernatant, dimethyl sulfoxide (DMSO; 150 μL/well) was added into each well to dissolve the formazan crystals. The absorbance was measured using a microplate reader at 570 nm with a 630-nm reference. The percentage (%) viability was calculated based on the following formula: % = absorbance value of treated cells/ absorbance value of vehicle-treated cells.

### Apoptosis assay by flow cytometry

To detect cellular apoptosis, cells were dual stained with Annexin V and propidium iodide (PI) (50 μg/mL; BD Biosciences, San Jose, CA, USA) following the manufacturer’s instructions and detected by Cytoflex Flow Cytometer (Beckman Coulter, Brea, CA, USA). The percentage (%) of cells with DNA contents representing the subG1, G0/G1, S, and G2/M phase was analyzed using EXPO32 ADC software (Beckman Coulter).

### Western blot

AMs were collected and lysed using cell lysis buffer (Beyotime, China). Equal amount of total proteins from each sample was separated on SDS-PAGE gel, and blotted onto a polyvinylidene difluoride membrane. The target protein was probed with one of the following primary antibodies (all from Cell Signaling Technology, Danvers, MA, USA) at 4 °C overnight: anti-LC3-I, anti-LC3-II, anti-BECN1, anti-SQSTM1/p62, anti-Bcl-2, anti-Bax, anti-cleaved caspase 3, anti-cleaved caspase 8, anti-cleaved caspase 9, anti-cleaved PARP, anti-MAPK1, anti-p-MAPK1, anti-MAPK8, anti-p-MAPK8, or anti-GAPDH (internal control). After the incubation with horseradish peroxidase-conjugated secondary antibodies at room temperature for 2 h, the signal was developed using the ECL system according to the manufacturer’s instructions. The signal density was analyzed using NIH Image J software and the relative protein level was calculated as the density ratio of the target protein to GAPDH (internal control).

### Immunofluorescence staining

The detection of LC3-II in phagosome membrane was performed by immunofluorescence, as described previously [20]. Briefly, cells grown on glass coverslips were treated as indicated, fixed with cold 100% methanol for 5 min, and washed with PBS. After blocking in antibody dilution solution (Abdil-Tx; TBS containing 0.1% Triton X-100, 2% BSA, and 0.1% sodium azide) at room temperature for 30 min, cells were incubated in anti-LC3-II antibody (1:1000) diluted in Abdil-Tx at 4 °C overnight, washed three times, incubated with fluorophore-conjugated secondary antibody. The coverslips were mounted onto glass slides using DAPI mounting medium (Vector Laboratories, CA, USA), imaged under the Olympus IX83 microscope (Tokyo, Japan), and the percentage (%) of LC3-II-positive cells or LC3-II^+^SQSTM1^+^ cells of all DAPI^+^ cells was calculated and averaged from at least five random images per sample.

### ALI rat model

The LPS-induced septic ALI model was established as described previously [21]. Briefly, rats were anesthetized with an intraperitoneal injection of 4 mL/kg body weight of a mixture of ketamine (20 mg/mL) and thiazines (2 mg/mL) and randomly divided into five groups (*n* = 6/group) to receive the following one or two-step instillations: PBS (control group), BML-111 (1 mg/kg body weight; BML-111 group), LPS (5 mg/kg body weight; ALI group), PBS + LPS (5 mg/kg body weight; PBS + ALI group), or BML-111 + LPS (BML-111 + ALI group). For each step, the total volume of the instillation was 100 μL, which was administered into the trachea using a syringe equipped with a blunt-end needle. The first instillation was followed by a waiting period of 1 h before the second one administered. After the instillation from each step, the rats were ventilated mechanically with 0.8 mL air for three times to allow equal distribution of the drugs. At 8 h after the second instillation, all rats were sacrificed, and the lung tissue was excised and immediately measured for its weight (wet weight, W). The lung tissue was then dried at 60 °C for five days and weighted again for dry weight (D). The W/D ratio was then calculated as an index of lung edema.

### Hematoxylin and eosin (HE) staining

The isolated lung tissues were fixed in 4% paraformaldehyde at room temperature for 24 h, washed with PBS, and embedded in paraffin. Sections of 4-μm in thickness were made and stained with hematoxylin and eosin (Vector Laboratory) following the manufacturer’s instructions. An ALI score was generated based on five independent features observed from HE images: neutrophils in the alveolar space, neutrophils in the interstitial space, hyaline membranes, proteinaceous debris filling the airspaces, and alveolar septal thickening, as described previously [22].

### Enzyme-linked immunosorbent assay (ELISA)

Bronchoalveolar lavage (BAL) was collected from each rat after three suctions, as described before [23]. The levels of TNF-α and IL-6 in BAL were measured using the ELISA kits for the corresponding cytokines (R&D Systems, Minneapolis, MN, USA) following the manufacturer’s instructions.

### Reverse transcription followed by quantitative real-time PCR (qRT-PCR)

Total RNA was extracted from isolated AMs using Trizol reagent (Invitrogen, Carlsbad, CA, USA), following the manufacturer’s instructions. cDNA was then synthesized using Takara reverse transcription system (Dalian, China). Quantitative PCR analysis was performed on ABI-7500 using iQTM SYBR® Green Supermix (Bio Rad, Hercules, CA; Cat# 170–3884) reagent. The following primers were used in this study: TNFα forward primer 5′- TGACAAGCCTGTAGCCCACG-3′, reverse primer 5′- TTGTCTTTGAGATCCATGCCG-3′; IL-6 forward primer 5′- TTCCATCCAGTTGCCTTCTT-3′, reverse primer 5’-CAGAATTGCCATTGCACAAC-3′; GAPDH (internal control) forward primer 5’-AGCCCAAGATGCCCTTCAGT-3′, reverse primer 5′- CCGTGTTCCTACCCCCAATG-3′. The relative expression of a target gene to that of the internal control was calculated using the 2^-ΔΔCt^ method [24].

### Statistical analysis

Quantitative data from in vitro experiments were presented as mean ± SD from at least three independent experiments. All data were analyzed by SPSS 13.0 software (IBM, Armonk, NY, USA). Differences between groups were assessed by one-way ANOVA with Tukey’s post-hoc analysis. *P* ≤ 0.05 was considered statistically significant.

## Results

### BML-111 inhibited LPS-induced apoptosis in AMs

**Fig. 1:**
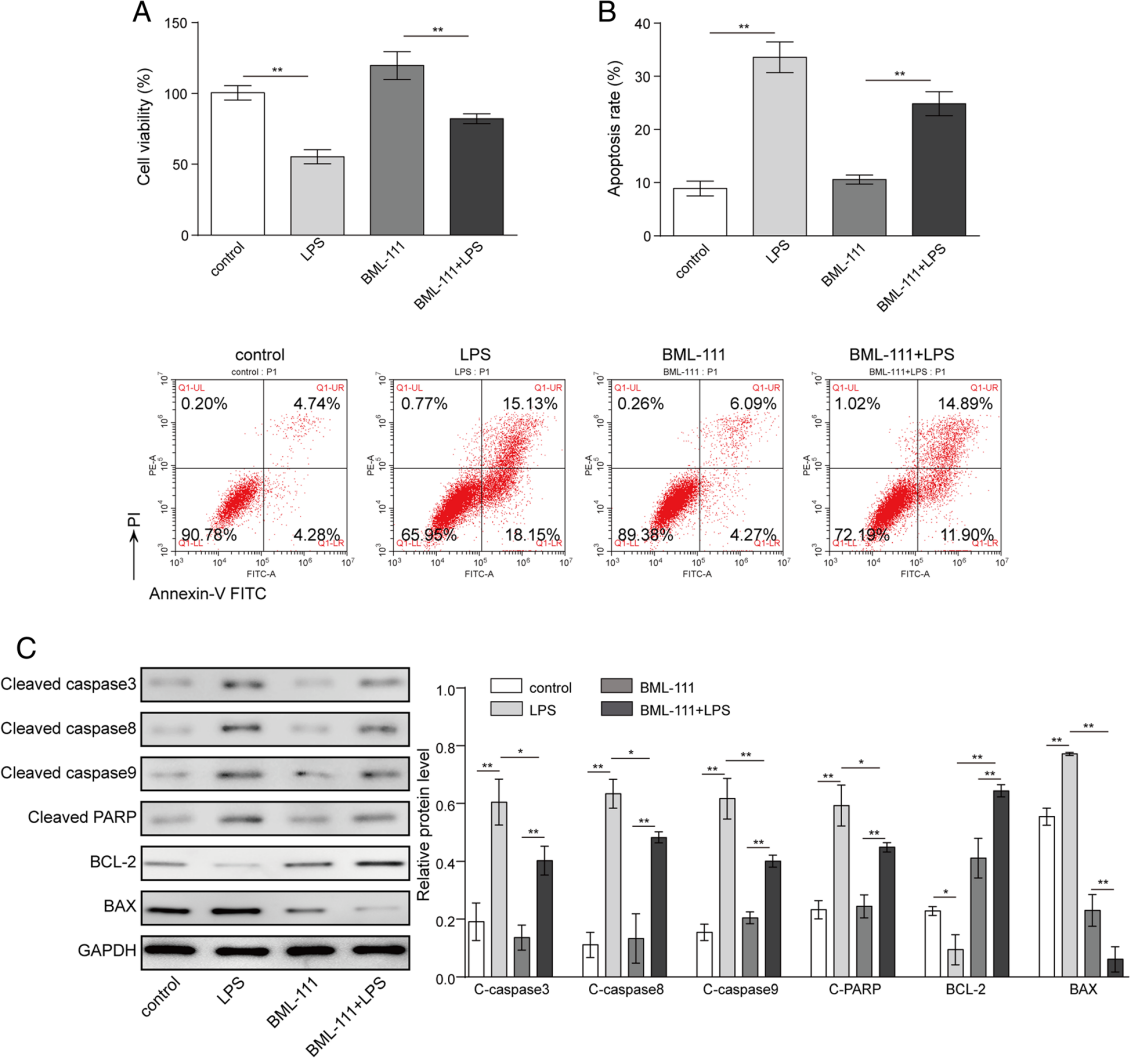
BML-111 inhibited LPS-induced apoptosis. AM were isolated from rats and treated with either vehicle (PBS), LPS (to induce ALI), BML-111, BML-111 + LPS. **a** At 24 h after the treatment, the cell viability was examined by MTT assay. **b** The apoptosis of cells was determined by flow cytometry following staining the cells with Annexin V and PI. **c** The expression of different apoptosis biomarkers, including cleaved-Caspase 3, cleaved-Caspase 8,cleaved-Caspase 9, cleaved-PARP, Bcl-2, and Bax was detected by Western blot. Representative Western blot image was shown on the left and the quantification of each protein level relative to that of the internal control (GAPDH) shown on the right. *n* = 3, *P < 0.05, **P < 0.01

LPS is a most commonly used and well-characterized inducer for experimental ALI. To assess whether lipoxin A4 confers any preventative benefits in LPS-induced ALI, we focused on AMs, isolated these cells from rats, pre-treated them with BML-111 for 6 h, and then challenged them with LPS. By MTT assay, we found that LPS significantly reduced cell viability (*P* < 0.05, comparing control- to LPS-treated cells), as expected (Fig. 1A). While BML-111 alone did not significantly affect the viability of normal AMs (*P* > 0.05, comparing control- to BML-111 treated cells), pre-treating AMs with BML-111 potently boosted the viability of LPS-treated cells (*P* < 0.05, comparing LPS- to BML-111 + LPS-treated cells; Fig. 1a). When monitoring AMs for apoptosis by dual staining with Annexin V and PI, we observed that LPS potently induced apoptosis, increased apoptosis rate from an average of 9.02% in control PBS-treated cells to approximately 33.28% in LPS-treated cells (*P* < 0.05); the latter was partially yet significantly reduced by the pre-treatment of cells with the lipoxin A4 agonist BML-111 (*P* < 0.05, comparing LPS- to BML-111 + LPS-treated cells), even though BML-111 alone did not significantly affect cellular apoptosis (*P* > 0.05, comparing control- to BML-111-treated cells; Fig. 1b). In addition, we also measured the changes of apoptosis-related proteins (Fig. 1c), including cleaved caspase 3, cleaved caspase 8, cleaved caspase 9, cleaved PARP and Bax, and anti-apoptotic Bcl-2. LPS significantly upregulated the expression of all pro-apoptotic proteins and downregulated that of anti-apoptotic Bcl-2, while BML-111 robustly inhibited the increase of pro-apoptotic proteins and elevated the level of Bcl-2. Taken together, these data suggest that when applied as a pre-treatment, BML-111 significantly and specifically antagonized the effects of LPS on the viability and apoptosis of AMs.

### BML-111 promoted autophagy in AMs

**Fig. 2:**
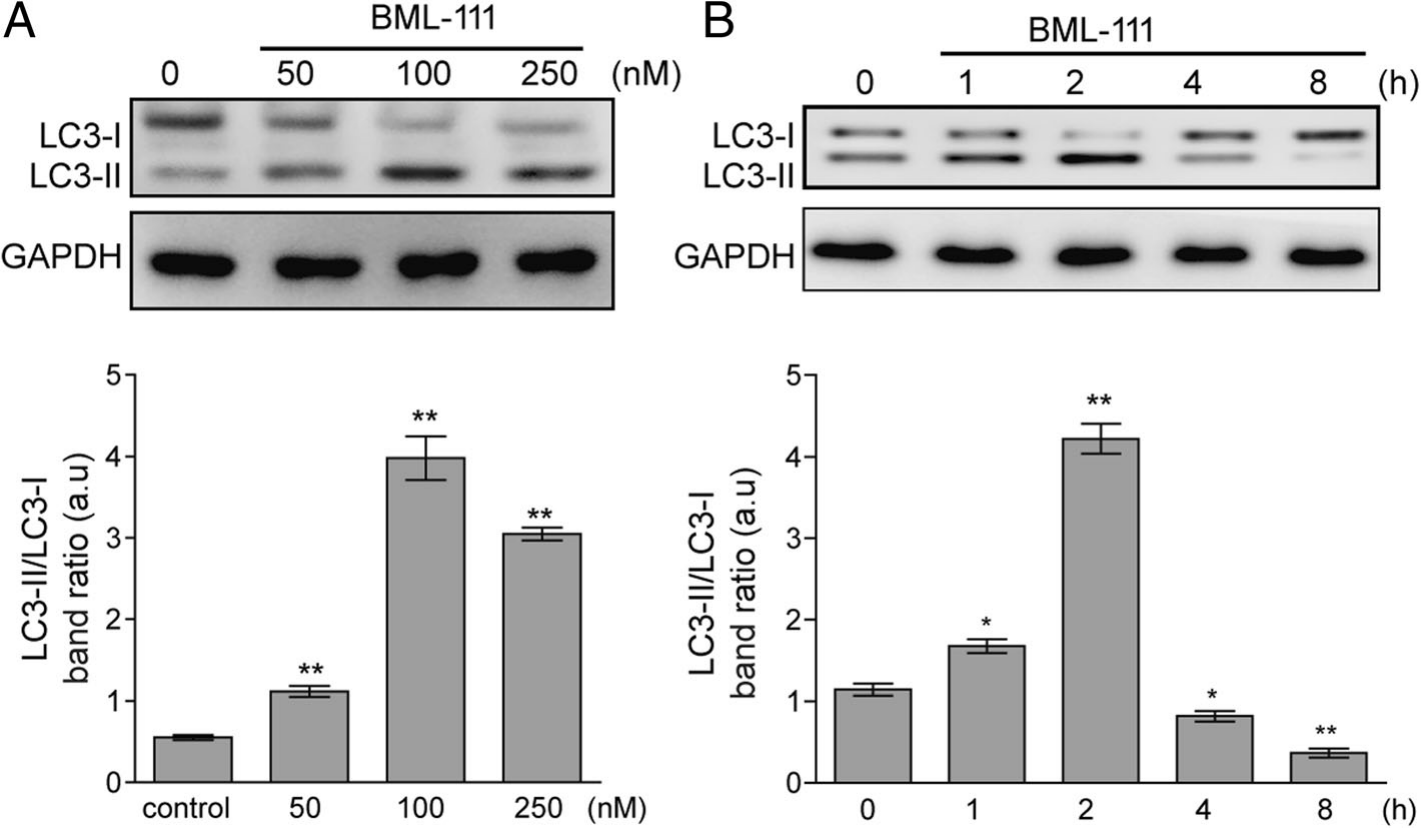
BML-111 elevated LC3-II level in AMs. AMs were treated with increasing concentrations of BML-111 for 2 h (**a**) or with 100 nM of BML-111 for indicated time periods (**b**). The expression of LC3-I and LC3-II was examined by Western blot. Representative Western blot image was shown on the top and the LC3-II/LC3-I ratio shown on the bottom. *n* = 3, *P < 0.05, **P < 0.01

Autophagy is critical for macrophage survival and LXs importantly regulate autophagy [25]. Therefore, we further assessed the effect of BML-111 on the autophagy of normal AMs. In response to increasing concentrations of BML-111, the autophagy reached peak at 100 nM BML-111 (*P* < 0.01, when compared to control cells; Fig. 2a), as represented by the highest LC3-II/LC3-I ratio. Under this optimal concentration of BML-111, the time-course study revealed that treatment with BML-111 for 2 to 4 h induced the highest level of autophagy (*P* < 0.01, when compared to control cells; Fig. 2b). Taken together, the data suggest that BML-111 is sufficient to promote autophagy of AMs and thus may protect these cells from LPS-induced apoptosis.

### BML-111 elevated autophagy level in LPS-treated AMs

**Fig. 3:**
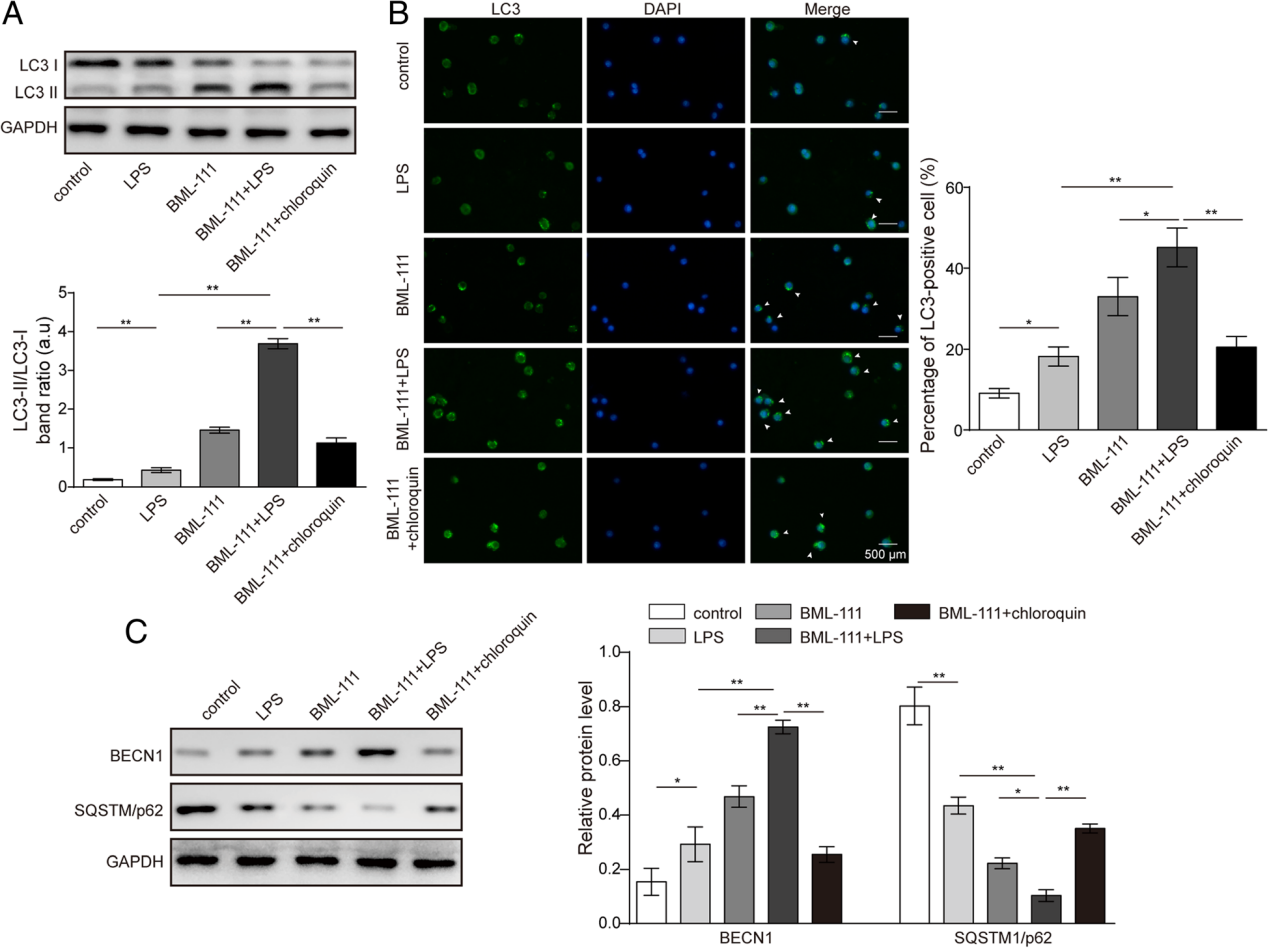
BML-111 elevated autophagy level in LPS-treated AM. AMs were treated as indicated. **a** The expression of LC3-I and LC3-II was examined by Western blot. Representative Western blot image was shown on the top and the LC3-II/LC3-I ratio shown on the bottom. **b** The expression of LC3-II in AM was detected by immunofluorescence (green signal). All cells were counter stained with DAPI (blue signal). Representative immunofluorescence images from indicated cells were shown on the left and the percentage of LC3-II+ cells quantified and shown as histogram on the right. **c** The expression of different autophagy and apoptosis biomarkers, including BECN1, SQSTM1/p62 was detected by Western blot. *n* = 3, *P < 0.05, **P < 0.01

The effect of BML-111 in normal AMs prompted us to examine its role in autophagy under ALI conditions. As shown in Fig. 3a, LPS minimally affected, yet BML-111 alone potently elevated the LC3-II level and thus the LC3-II/LC3-I ratio (*P* < 0.05, when comparing LPS-treated or BML-111-trated cells with control cells). The highest LC3-II/LC3-I ratio was achieved in cells pre-treated with BML-111 followed by LPS (BML-111 + LPS; *P* < 0.05, when compared to all other groups). Consistently, immunofluorescence staining revealed that BML-111 alone was sufficient to increase intracellular LC3-II level, while the highest LC3-II level was present in cells treated with BML-111 + LPS (Fig. 3b). In addition to LC3, we examined the effects of BML-111 on the level of two autophagy-related proteins BECN1 and SQSTM1/p62. Western blot showed that the level of BECN1 was significantly up-regulated, while that of SQSTM1/p62 down-regulated by BML-111 and more robustly by LPS + BML-111 (Fig. 3c). To address that the increased LC3-II/LC3-I ratio and the elevated BECN1 and SQSTM1/p62 were due to enhanced autophagy but not reduced autophagosome degradation, we added chloroquine, an autophagy inhibitor, to BML-111-challenged cells (BML-111 + chloroquine). As shown in Figs. 3a to 3c, the LC3-II/LC3-I ratio, LC3 and BECN1 levels were significantly lower, while SQSTM1/p62 level markedly higher in BML-111 + choloquine cells than in BML-111 alone cells, supporting the specific effects of BML-111 on autophagy. Taken together, these data suggest that BML-111, when applied as a pre-treatment to LPS-treated macrophages, significantly boosted autophagy.

### BML-111 targeted MAPK pathway but not mTOR signaling to induce autophagy

**Fig. 4:**
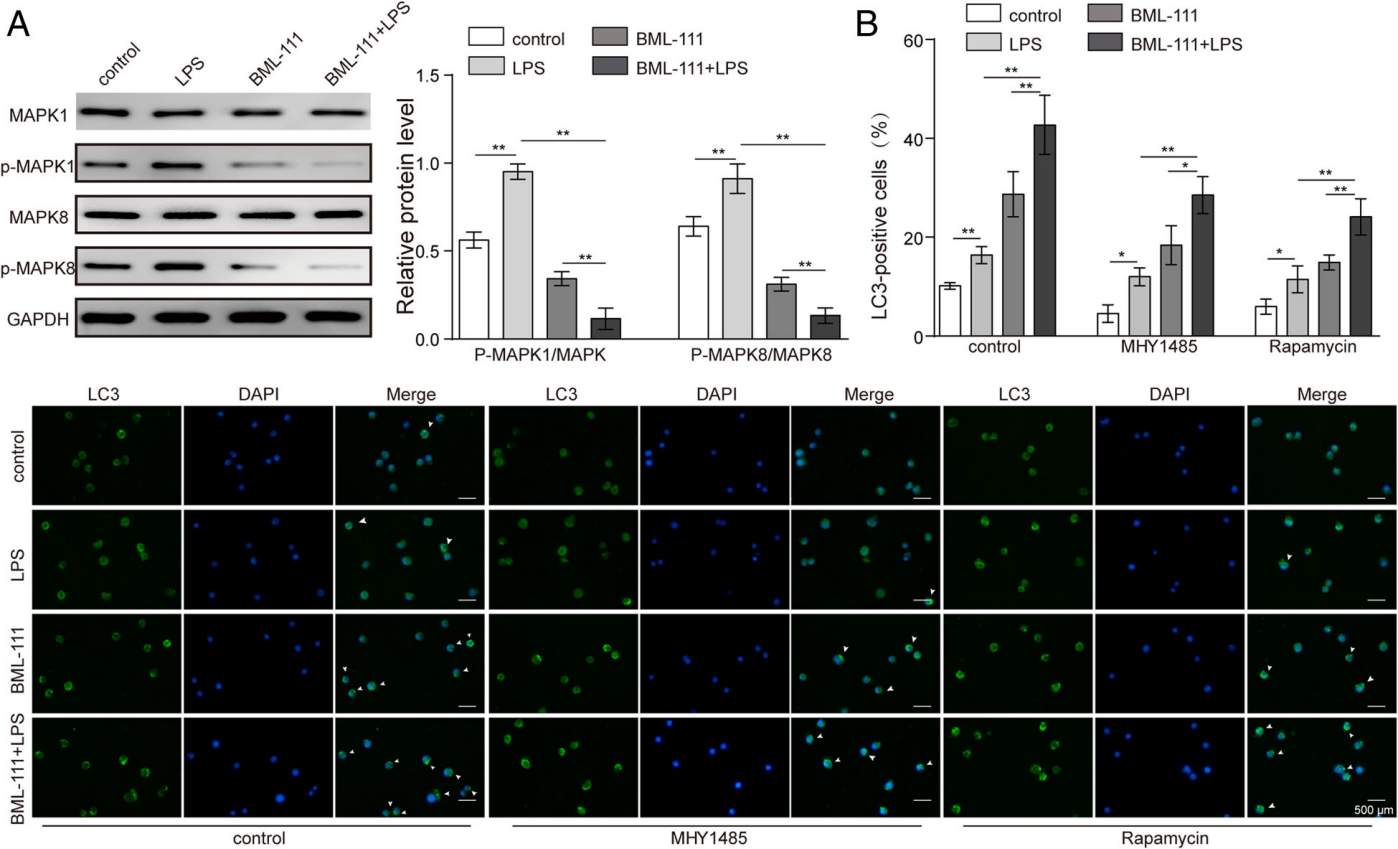
BML-111 targeted MAPK1 pathway but mTOR-independent mechanism to induce autophagy. **a** The activation of MAPK1 and MAPK8 was detected by Western blot in AM treated as indicated. Representative Western blot image was shown on the left and the quantification of each protein level relative to that of the internal control (GAPDH) shown on the right. **b** AM were treated as indicated, in autophagy inhibitor MHY-1485 and mTOR inhibitor Rapamycin. LC3-II expression was examined by immunofluorescence (green signals). All cells were counter stained with DAPI (blue signal). Representative immunofluorescence images from indicated cells were shown on the bottom and the percentage of LC3-II+ cells quantified and shown as histogram on the top. *n* = 3, *P < 0.05, **P < 0.01

Both MAPK and mTOR pathways play significant roles in regulating autophagy [18, 26]. By examining the status of MAPK1 and MAPK8 in AMs in response to BML-111 and/or LPS, we found that LPS alone significantly boosted, while BML-111 alone potently inhibited the activation of both MAPK1 and MAPK8 (*P* < 0.05, when compared to all other groups; Fig. 4a). The most robust suppression in MAPK1 and MAPK8 was achieved in BML-111 + LPS cells (*P* < 0.05; when compared to all other groups), suggesting that BML-111 pre-treatment significantly and specifically targeted MAPK pathway in response to LPS.

To assess the significance of mTOR signaling in BML-111-induced autophagy, we treated cells with either MHY-1485, a well-demonstrated mTOR activator [27], or rapamycin, the classical mTOR inhibitor. We found that neither MHY-1485 nor rapamycin noticeably affected the % of LC3^+^ cells in cells treated with BML-111 and/or LPS (Fig. 4b), suggesting that the mTOR signaling does not participate in BML-111-induced autophagy.

### BML-111 alleviated ALI in vivo

**Fig. 5:**
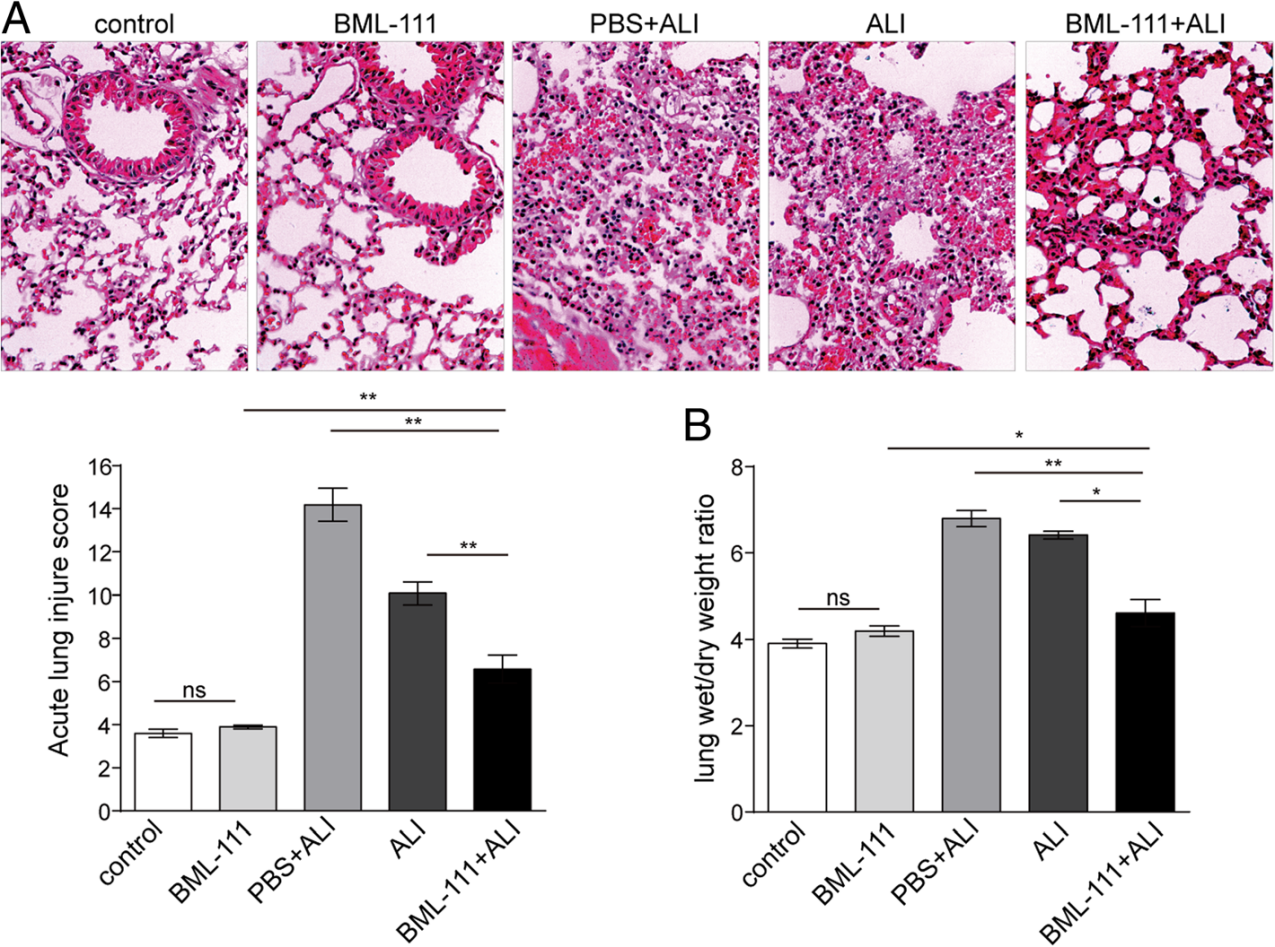
BML-111 alleviated ALI in vivo. ALI model was established in rats by intratracheal instillation of LPS and rats were either not treated (ALI), or treated with vehicle (PBS + ALI) or BML-111 (BML-111 + ALI). As controls, rats not through ALI induction and treated with either vehicle (PBS) or BML-111 were used. **a** Upon sacrifice, the lung tissue from each group was examined by HE staining and assessed for ALI score. **b** The lung tissue was measured for wet/dry weight ratio and compared among different groups. *n* = 6, *P < 0.05, **P < 0.01

AMs are the central player in resolving inflammation and initiating tissue repair for ALI [4]. Considering that BML-111 pre-treatment could induce autophagy and inhibit apoptosis in LPS-treated AMs (Figs. 1-4), we want to examine whether BML-111 may preventatively benefit ALI in vivo*.* In control rats or rats receiving BML-111 alone, no significant lung injury was noticed. In contrast, we found extensive inflammation and lung injury in the lung tissues from ALI rats or ALI + PBS (vehicle) rats, which were dramatically alleviated in lungs from BML-111 + ALI rats (*P* < 0.05, Fig. 5a). Consistently, the score of acute lung injury (Fig. 5A) and lung wet/dry weight ratio (Fig. 5b) were significantly higher in ALI rats than in BML-111 + ALI rats, suggesting that the prophylactic administration of BML-111 robustly alleviates ALI-associated lung injury.

### The benefits of BML-111 were associated with reduced inflammation and enhanced autophagy in vivo

**Fig. 6:**
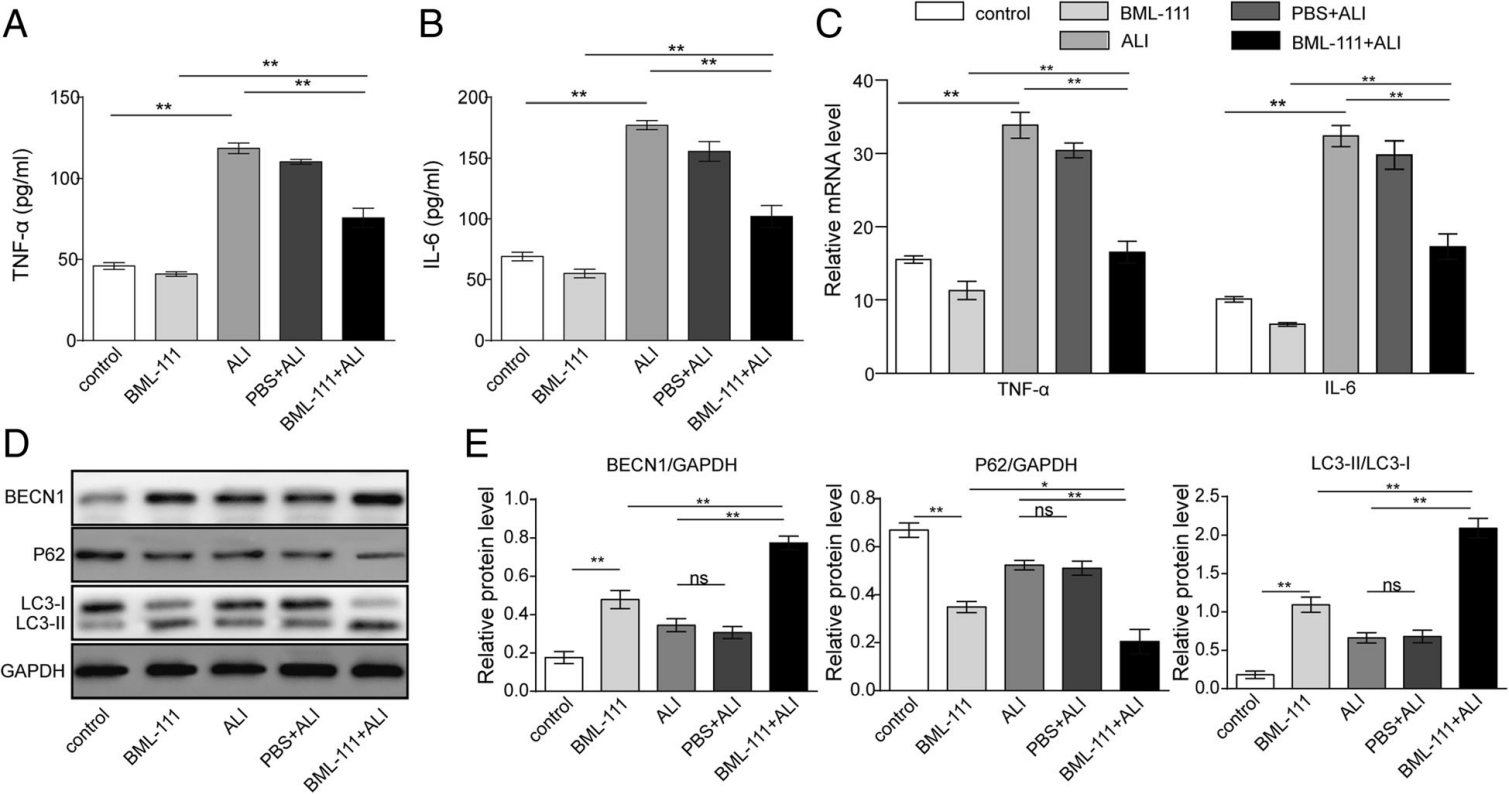
The benefits of BML-111 were associated with reduced inflammation and enhanced autophagy in vivo. Bronchoalveolar lavage was collected from rats of each group and the levels of TNF-α (**a**) and IL-6 (**b**) was measured using ELISA. AMs were isolated from rats of each group. **c** The expressions of TNF-α and IL-6 on the steady-state mRNA level were measured by RT-qPCR. **d** The expressions of BECN1, SQSTM1/p62, LC3-I, and LC3-II in isolated AM were examined by Western blot. Representative Western blot image was shown on the left and the quantification of each protein level relative to that of the internal control (GAPDH) shown on the right (**e**). n = 6, *P < 0.05, **P < 0.01

To explore the molecular mechanisms underlying the preventative benefits of BML-111 in vivo (Fig. 5), we first measured the pro-inflammatory cytokines, TNF-α and IL-6 from BAL. In ALI rats, the levels of both cytokines were dramatically up-regulated in the lung lavage (*P* < 0.01, when compared to control or BML-111 rats). The pre-administration of BML-111 into rats induced for ALI significantly reduced the levels of both cytokines (*P* < 0.01, Fig. 6a and Fig. 6b). Consistently, the mRNA levels of both cytokines in isolated AMs from each group showed the same trend as their protein levels in BAL (*P* < 0.01, Fig. 6c), suggesting that BML-111 pre-treatment significantly resolved lung inflammation. Furthermore, Western blot showed that autophagy, as represented by the level of BECN1, SQSTM1/p62, LC3-I, and LC3-II, was significantly activated in isolated AMs from BML-111-pre-treated ALI rats, when compared to ALI rats (*P* < 0.01, Fig. 6d), supporting contribution of BML-111-induced autophagy to ALI amelioration.

## Discussion

To date, optimal treatment strategy for ALI is not established and clinical practice mainly centers on supportive ventilatory treatment and conservative fluid management [28]. Increasing understanding on the pathophysiology of ALI has resulted in various pharmacologic therapies, such as surfactants, nitric oxide, corticosteroids, etc., which although presenting promising pre-clinical effects, have not shown equal success in clinical trials [28]. In this study, we used an in vivo ALI rat model and presented pre-clinical evidence that lipoxin A4 receptor agonist BML-111, when applied preventatively, significantly and specifically alleviated ALI. More importantly, when focusing on AMs, we showed that BML-111 induced autophagy and inhibited apoptosis of these cells, suppressing inflammation and ameliorating lung injury.

The central but dichotomous roles of AMs in orchestrating the progression of ALI present these cells as an ideal yet challenging target for ALI treatment. Concomitant with the disease progression from an early inflammatory to the late resolution phase, AMs coordinately transition from the pro-inflammatory M1 phenotype to the anti-inflammatory M2 state [29]. Understanding the mechanisms regulating the phenotypic transition of AMs will surely help to develop dual-targeting therapies, i.e. simultaneously alleviating inflammation and promoting tissue repair. Although these mechanisms largely remain elusive for ALI, studies suggest that AMs are an important source of LXs, and the increase of LXs within the pulmonary microenvironment promotes apoptosis of neutrophils and at the same time enhances the phagocytosis/clearance of apoptotic neutrophils by macrophages, presenting dual anti-inflammatory and pro-resolution activities [4, 30]. Failure to completely remove neutrophils from the lesion and return the tissue to homeostasis resulted in chronic inflammation and fibrosis. Therefore, LXs have been widely examined as therapeutic agents for inflammation-related pathologies, such as cancer [31, 32], arthritis [33], asthma [33], and cardiovascular diseases [34]. The actions of LXs in target cells are mediated through the LXA4 receptors. Due to the short lifespan of endogenous LXs, stable LX analogs or LXA4 receptor agonists are designed and intensively examined in various studies. Here we used BML-111, a LXA4 receptor agonist to investigate the mechanisms and therapeutic potential of LXs in ALI pathogenesis.

Several studies have shown the pleotropic effects of LXs on ALI, which are achieved by targeting distinct cell populations within the pulmonary tissue. Cheng et al. reported that by LXA4 up-regulated Nrf2-mediated E-cadherin expression in alveolar epithelial cells, preserved airway permeability, and attenuated LPS-induced ALI [14]. Mesenchymal stem cells presented therapeutic benefits to ALI, which was mediated at least partially through LXA4 receptor [35]. Aspirin-induced 15-epi-LXA4 boosts the expression of heme oxygenase-1, prevents the formation of neutrophil-platelet aggregation, and thus attenuates ALI [36, 37]. 15-epi-LXA4 promotes neutrophil apoptosis by suppressing the expression of myeloperoxidase [38]. By inhibiting pro-inflammatory NF-κB and p38 MAPK signaling pathways and elevating the expression of heme oxygenase-1 in endothelial cells, LXA4 protected pulmonary endothelial cells from TNF-α-induced inflammatory damages [15]. In this study, we added a novel mechanism to the repertoire of protective activities of LXs as a prophylactic reagent during ALI development, i.e. to induce autophagy and inhibit apoptosis of AMs, promoting the survival of these cells and reducing inflammatory injuries.

Autophagy and apoptosis are two critical yet interrelated biological processes controlling the phenotypes and functions of macrophages. In macrophages, autophagy may contribute to cell death by promoting apoptosis or when apoptosis is blocked [39, 40]; under other circumstances, however, autophagy provides a survival mechanism that protects cells from apoptosis and enable them to achieve other functions, such as differentiation and polarization [5, 11, 41]. Consistent with the second scenario, here we showed that BML-111 simultaneously induced autophagy and reduced apoptosis in AMs, leading to enhanced survival and dampened inflammatory responses, as represented by the reduced production of pro-inflammatory cytokines TNFα and IL-6. The induction of autophagy is not unique for ALI-induced AMs, since BML-111 is sufficient to activate autophagy even in cells under homeostasis. It is also noted that activation of autophagy is not a novel bioactivity identified for LXs. Borgeson et al. reported that LXA4 alleviated obesity-induced adipose inflammation, which was associated with the transition of macrophages within the adipose tissue from M1 to M2 phenotypes, as well enhanced autophagy of adipose tissue [42]. Prieto et al. showed that 15-epi-LXA_4_ promoted autophagy in both murine and human macrophages, through the activation of MAPK1 and NFE2L2 pathways and independent of mTOR signaling, leading to improved survival and phagocytosis of these cells [18]. Although we identified similar functional consequences in ALI-induced AM upon BML-111 pre-treatment, we showed that the activation of both MAPK1 and MAPK8 was suppressed by BML-111, supporting its importance in BML-111-induced autophagy. In another study, LXA4 inhibited apoptosis of macrophages by activating PI3K/Akt and ERK/Nrf-2 pathways [43]. Considering the complex network regulating autophagy (both mTOR-dependent and mTOR-independent) and apoptosis [44], it is important to follow up on this study to further dissecting the signaling cascades mediating BML-111-activated autophagy and inhibited apoptosis, which will reveal potential targets that could shift the balance of AMs from ALI-induced apoptosis to autophagy.

Although enhanced autophagy in AMs by BML-111 from this study was associated with reduced inflammation and alleviated ALI, it is not known whether such association is attributed to the phenotype transition of macrophages from M1 to M2. Impaired autophagy in macrophages led to proinflammatory polarization and exacerbated immune response in obese mice [45], while selective autophagy may promote the polarization to M2 phenotype [46]. It is therefore critical to characterize the phenotypes of ALI-induced AM in response to BML-111 treatment. More importantly, we should comprehensively profile the differences in signaling mechanisms as well as biological functions of AM before and after BML-111 treatment, in order to identify the critical signaling molecules that control the phenotypic and functional transition of these cells from pro-inflammation to pro-resolution.

## Conclusion

In summary, we provide pre-clinical evidence that LXA4 receptor agonist BML-111 presents prophylactic benefits of ALI. On the cellular level, BML-111 activates autophagy and inhibits apoptosis of AMs, promoting their survival and alleviating pulmonary inflammation in response to ALI challenge. Although this study focuses on the preventative effects of BML-111, the data suggest that BML-111 may also act on the same signaling pathways and provide therapeutic advantages for ALI, which should be further explored using proper cell culture systems as well as ALI-related animal models.

## Abbreviations

ALI: acute lung injury
AMPK: AMP activated protein kinase
AMs: alveolar macrophages
ARDS: acute respiratory distress syndrome
BECN1: Beclin 1
ELISA: enzyme-linked immunosorbent assay
HE: hematoxylin and eosin
IFN: interferon
IL: interleukin
LC3-I: cytosolic form of LC3
LC3-II: LC3-phosphatidylethanolamine conjugate
LPS: lipopolysaccharide
LXs: lipoxins
MAPK1: mitogen-activated protein kinase1
MAPK8: mitogen-activated protein kinase8
mTOR: *mammalian target of rapamycin*
MTT: 3-(4,5-dimethylthiazol-2-yl)-2,5-diphenyltetrazolium bromide
PI: propidium iodide
qRT-PCR: *quantitative real-time PCR*
TNFα: tumor necrosis factor α

## References

[1] Thompson BT, Chambers RC, Liu KD (2017). Acute respiratory distress syndrome. N Engl J Med.

[2] Matthay MA, Zemans RL (2011). The acute respiratory distress syndrome: pathogenesis and treatment. Annu Rev Pathol.

[3] Zhao M, Fernandez LG, Doctor A, Sharma AK, Zarbock A, Tribble CG, Kron IL, Laubach VE (2006). Alveolar macrophage activation is a key initiation signal for acute lung ischemia-reperfusion injury. Am J Physiol Lung Cell Mol Physiol.

[4] Herold S, Mayer K, Lohmeyer J (2011). Acute lung injury: how macrophages orchestrate resolution of inflammation and tissue repair. Front Immunol.

[5] Chen P, Cescon M, Bonaldo P (2014). Autophagy-mediated regulation of macrophages and its applications for cancer. Autophagy.

[6] Mizushima N (2007). Autophagy: process and function. Genes Dev.

[7] Kim J, Kundu M, Viollet B, Guan KL (2011). AMPK and mTOR regulate autophagy through direct phosphorylation of Ulk1. Nat Cell Biol.

[8] M. Kadowaki, M.R. Karim, Cytosolic LC3 ratio as a quantitative index of macroautophagy, Methods Enzymol 452 (2009) 199–213. 10.1016/S0076-6879(08)03613-6 19200884

[9] Kang R, Zeh HJ, Lotze MT, Tang D (2011). The Beclin 1 network regulates autophagy and apoptosis. Cell Death Differ.

[10] Komatsu M, Ichimura Y (2010). Physiological significance of selective degradation of p62 by autophagy. FEBS Lett.

[11] Fan T, Chen L, Huang Z, Mao Z, Wang W, Zhang B, Xu Y, Pan S, Hu H, Geng Q (2016). Autophagy decreases alveolar macrophage apoptosis by attenuating endoplasmic reticulum stress and oxidative stress. Oncotarget.

[12] Hu R, Chen ZF, Yan J, Li QF, Huang Y, Xu H, Zhang X, Jiang H (2014). Complement C5a exacerbates acute lung injury induced through autophagy-mediated alveolar macrophage apoptosis. Cell Death Dis.

[13] Chandrasekharan JA, Sharma-Walia N (2015). Lipoxins: nature's way to resolve inflammation. J Inflamm Res.

[14] Cheng X, He S, Yuan J, Miao S, Gao H, Zhang J, Li Y, Peng W, Wu P (2016). Lipoxin A4 attenuates LPS-induced mouse acute lung injury via Nrf2-mediated E-cadherin expression in airway epithelial cells. Free Radic Biol Med.

[15] Lv W, Lv C, Yu S, Yang Y, Kong H, Xie J, Sun H, Andersson R, Xu D, Chen B, Zhou M (2013). Lipoxin A4 attenuation of endothelial inflammation response mimicking pancreatitis-induced lung injury. Exp Biol Med (Maywood).

[16] Bannenberg G, Moussignac RL, Gronert K, Devchand PR, Schmidt BA, Guilford WJ, Bauman JG, Subramanyam B, Perez HD, Parkinson JF, Serhan CN (2004). Lipoxins and novel 15-epi-lipoxin analogs display potent anti-inflammatory actions after oral administration. Br J Pharmacol.

[17] Zhang L, Zhang X, Wu P, Li H, Jin S, Zhou X, Li Y, Ye D, Chen B, Wan J (2008). BML-111, a lipoxin receptor agonist, modulates the immune response and reduces the severity of collagen-induced arthritis. Inflamm Res.

[18] Prieto P, Rosales-Mendoza CE, Terron V, Toledano V, Cuadrado A, Lopez-Collazo E, Bannenberg G, Martin-Sanz P, Fernandez-Velasco M, Bosca L (2015). Activation of autophagy in macrophages by pro-resolving lipid mediators. Autophagy.

[19] Signarovitz AL, Ray HJ, Yu JJ, Guentzel MN, Chambers JP, Klose KE, Arulanandam BP (2012). Mucosal immunization with live attenuated Francisella novicida U112DeltaiglB protects against pulmonary F. tularensis SCHU S4 in the Fischer 344 rat model. PLoS One.

[20] Zhang X, Liu Q (2013). Autophagy assays (LC3B immunofluorescence, LC3B western blot, acridine orange assay). Bio-protocol.

[21] Shimizu M, Hasegawa N, Nishimura T, Endo Y, Shiraishi Y, Yamasawa W, Koh H, Tasaka S, Shimada H, Nakano Y, Fujishima S, Yamaguchi K, Ishizaka A (2009). Effects of TNF-alpha-converting enzyme inhibition on acute lung injury induced by endotoxin in the rat. Shock.

[22] Matute-Bello G, Downey G, Moore BB, Groshong SD, Matthay MA, Slutsky AS, Kuebler WM, Acute Lung G (2011). Injury in animals study, an official American Thoracic Society workshop report: features and measurements of experimental acute lung injury in animals. Am J Respir Cell Mol Biol.

[23] Song JA, Yang HS, Lee J, Kwon S, Jung KJ, Heo JD, Cho KH, Song CW, Lee K (2010). Standardization of bronchoalveolar lavage method based on suction frequency number and lavage fraction number using rats. Toxicol Res.

[24] Rao X, Huang X, Zhou Z, Lin X (2013). An improvement of the 2^(−delta delta CT) method for quantitative real-time polymerase chain reaction data analysis. Biostat Bioinforma Biomath.

[25] Wu YT, Tan HL, Shui G, Bauvy C, Huang Q, Wenk MR, Ong CN, Codogno P, Shen HM (2010). Dual role of 3-methyladenine in modulation of autophagy via different temporal patterns of inhibition on class I and III phosphoinositide 3-kinase. J Biol Chem.

[26] Singh BN, Kumar D, Shankar S, Srivastava RK (2012). Rottlerin induces autophagy which leads to apoptotic cell death through inhibition of PI3K/Akt/mTOR pathway in human pancreatic cancer stem cells. Biochem Pharmacol.

[27] Zhu L, Hao J, Cheng M, Zhang C, Huo C, Liu Y, Du W, Zhang X (2018). Hyperglycemia-induced Bcl-2/Bax-mediated apoptosis of Schwann cells via mTORC1/S6K1 inhibition in diabetic peripheral neuropathy. Exp Cell Res.

[28] Johnson ER, Matthay MA (2010). Acute lung injury: epidemiology, pathogenesis. and treatment, J Aerosol Med Pulm Drug Deliv.

[29] Aggarwal NR, King LS, D'Alessio FR (2014). Diverse macrophage populations mediate acute lung inflammation and resolution. Am J Physiol Lung Cell Mol Physiol.

[30] Serhan CN, Chiang N, Van Dyke TE (2008). Resolving inflammation: dual anti-inflammatory and pro-resolution lipid mediators. Nat Rev Immunol.

[31] Chen Y, Hao H, He S, Cai L, Li Y, Hu S, Ye D, Hoidal J, Wu P, Chen X (2010). Lipoxin A4 and its analogue suppress the tumor growth of transplanted H22 in mice: the role of antiangiogenesis. Mol Cancer Ther.

[32] Janakiram NB, Rao CV (2009). Role of lipoxins and resolvins as anti-inflammatory and proresolving mediators in colon cancer. Curr Mol Med.

[33] Levy BD (2005). Lipoxins and lipoxin analogs in asthma. Prostaglandins Leukot Essent Fatty Acids.

[34] Vital SA, Becker F, Holloway PM, Russell J, Perretti M, Granger DN, Gavins FN (2016). Formyl-peptide receptor 2/3/Lipoxin A4 receptor regulates neutrophil-platelet aggregation and attenuates cerebral inflammation: impact for therapy in cardiovascular disease. Circulation.

[35] Fang X, Abbott J, Cheng L, Colby JK, Lee JW, Levy BD, Matthay MA (2015). Human mesenchymal stem (stromal) cells promote the resolution of acute lung injury in part through Lipoxin A4. J Immunol.

[36] Jin SW, Zhang L, Lian QQ, Liu D, Wu P, Yao SL, Ye DY (2007). Posttreatment with aspirin-triggered lipoxin A4 analog attenuates lipopolysaccharide-induced acute lung injury in mice: the role of heme oxygenase-1. Anesth Analg.

[37] Ortiz-Munoz G, Mallavia B, Bins A, Headley M, Krummel MF, Looney MR (2014). Aspirin-triggered 15-epi-lipoxin A4 regulates neutrophil-platelet aggregation and attenuates acute lung injury in mice. Blood.

[38] El Kebir D, Jozsef L, Pan W, Wang L, Petasis NA, Serhan CN, Filep JG (2009). 15-epi-lipoxin A4 inhibits myeloperoxidase signaling and enhances resolution of acute lung injury. Am J Respir Crit Care Med.

[39] Martyniszyn L, Szulc-Dabrowska L, Boratynska-Jasinska A, Struzik J, Winnicka A, Niemialtowski M (2013). Crosstalk between autophagy and apoptosis in RAW 264.7 macrophages infected with ectromelia orthopoxvirus. Viral Immunol.

[40] Xu Y, Kim SO, Li Y, Han J (2006). Autophagy contributes to caspase-independent macrophage cell death. J Biol Chem.

[41] Liao X, Sluimer JC, Wang Y, Subramanian M, Brown K, Pattison JS, Robbins J, Martinez J, Tabas I (2012). Macrophage autophagy plays a protective role in advanced atherosclerosis. Cell Metab.

[42] Borgeson E, Johnson AM, Lee YS, Till A, Syed GH, Ali-Shah ST, Guiry PJ, Dalli J, Colas RA, Serhan CN, Sharma K, Godson C (2015). Lipoxin A4 attenuates obesity-induced adipose inflammation and associated liver and kidney disease. Cell Metab.

[43] Prieto P, Cuenca J, Traves PG, Fernandez-Velasco M, Martin-Sanz P, Bosca L (2010). Lipoxin A4 impairment of apoptotic signaling in macrophages: implication of the PI3K/Akt and the ERK/Nrf-2 defense pathways. Cell Death Differ.

[44] Kim YC, Guan KL (2015). mTOR: a pharmacologic target for autophagy regulation. J Clin Invest.

[45] Liu K, Zhao E, Ilyas G, Lalazar G, Lin Y, Haseeb M, Tanaka KE, Czaja MJ (2015). Impaired macrophage autophagy increases the immune response in obese mice by promoting proinflammatory macrophage polarization. Autophagy.

[46] Chang CP, Su YC, Lee PH, Lei HY (2013). Targeting NFKB by autophagy to polarize hepatoma-associated macrophage differentiation. Autophagy.

